# Comparable clinical outcomes in functionally aligned computer‐assisted and image‐based robotic assisted total knee arthroplasty

**DOI:** 10.1002/ksa.70023

**Published:** 2025-08-29

**Authors:** Stefano Seracchioli, Francesco Zambianchi, Sebastiano Clemenza, Mattia Clò, Riccardo Cuoghi Costantini, Fabio Catani

**Affiliations:** ^1^ Department of Orthopaedics and Traumatology Azienda Ospedaliero‐Universitaria di Modena, University of Modena and Reggio‐Emilia Modena Italy; ^2^ Department of Medical and Surgical Sciences for Mother, Child and Adult University of Modena and Reggio‐Emilia Modena Italy; ^3^ Department of Orthopaedic Surgery School of Medicine, University of Pittsburgh Pittsburgh Pennsylvania USA

**Keywords:** clinical outcome, computer aided surgery, functional alignment, three‐dimensional image‐based robotic assisted, TKA

## Abstract

**Purpose:**

To determine the clinical outcomes differences and complications in two comparable groups of patients undergoing computer aided surgery (CAS) and robotic‐assisted (RA) posterior stabilised (PS) total knee arthroplasty (TKA) following functional alignment (FA) principles with tibial pre‐cut at a minimum of 4‐year follow‐up.

**Methods:**

This retrospective, monocentric and observational study included 94 consecutive patients undergoing PS TKA performed with CAS and RA‐TKA following FA principles, between January 2017 and January 2020. Patients were followed with radiological and clinical assessment and evaluated with the Forgotten Joint Score‐12 (FJS‐12), Knee Injury and Osteoarthritis Outcome Score for Joint Replacement (KOOS‐JR) and the 5‐Level Likert Scale (5‐LLS).

**Results:**

Out of 94 patients two were lost to follow‐up and one deceased in the robotic branch, eight patients were lost to follow‐up and two were deceased in the navigated branch. Hereby, a total of 84 patients (87 knees) with a mean age of 69.2 ± 8.4 years were considered. A total of 40 cases were included in the CAS group; 44 cases were included in the RA‐TKA group. No revisions were performed in any of the two groups, resulting in an overall Kaplan‐Meyer survivorship rate of 100% for both cohorts. At last follow‐up, no statistically significant differences were recorded between CAS and RA‐TKA relative to FJS‐12, KOOS‐JR and 5‐LLS (FJS‐12: 89.4 ± 9.5 vs. 88.3 ± 13.4; KOOS‐JR: 88.0 ± 10.2 vs 86.2 ± 11.5; 5‐LLS 4.4 ± 1.7 vs 4.6 ± 2.1) respectively.

**Conclusions:**

No significant outcomes differences and complications were detected between patients undergoing PS‐TKA performed with either CAS and RA at a minimum 4‐year follow‐up. TKA performed with a patient‐specific FA technique and with a soft tissue‐preserving approach, showed excellent results with both CAS and RA‐TKA.

**Level of Evidence:**

Level III.

**Abbreviations**
CAS: computer aided surgery
CPAK: coronal plane alignment of the knee
FA: functional alignment
FJS‐12: forgotten joint score‐12
JLO: Joint Line Obliquity
KOOS‐JR: Knee Injury and Osteoarthritis Outcome Score for Joint Replacement
LDFA: lateral distal femoral angle
MA: mechanical alignment
MCID: minimal clinical important difference
MPTA: medial proximal tibial angle
OA: osteoarthritis
PASS: patient acceptable symptom state
PS: posterior stabilised
RA: robotic‐assisted
TKA: total knee arthroplasty

## INTRODUCTION

Total knee arthroplasty (TKA) is the gold standard treatment for advanced osteoarthritis of the knee [6], yet a significant number of patients remain dissatisfied despite technically successful procedures [26]. A common reason for clinical dissatisfaction and TKA revision is related to soft tissue balancing of the knee, determining either stiffness or joint instability [17, 27]. To reduce such complications, computer aided surgery (CAS) and robotic‐assisted (RA) TKA have been introduced to improve accuracy or implant positioning, the precision of bone cuts, reduce the outliers in limb alignment, and achieve patient‐specific soft tissue balancing [32]. CAS has been applied to TKA for more than 20 years [5] but its use has remained peripheral in the context of TKA. This may be because with CAS implant positioning has historically been performed using mechanical alignment (MA) and a systematic approach. This has resulted in fewer radiologic outliers compared to conventional surgery [24] but has not translated into improved long‐term functional outcomes or implant survival, especially in long‐term follow‐up [14, 29] with the exception, reported by the Australian Arthroplasty Registry, of a reduced cumulative percentage of CAS TKA revision at 9‐year follow‐up in patients <65 years (*p*:0.01) [8]. In recent years, the introduction of robotics has been a breakthrough in TKA due to the ability to perform accurate cuts and implement several parameters including the amount of resections and components' alignment [15]. Such technology has contributed to the development and spread of a more “patient‐specific” alignment philosophy for TKA, including functional alignment (FA). FA is an individualised alignment strategy that aims to restore each patient's constitutional lower limb alignment and joint line orientation while minimising soft tissue releases. Bone resections and implant positioning are adapted based on the preoperative anatomy and intraoperative soft tissue tension, rather than applying systematic mechanical alignment targets. The surgical workflow prioritises balanced flexion and extension gaps while respecting the soft tissue of the knee [16, 25]. While both CAS [10] and RA can be used to apply FA principles, there is currently no study directly comparing their performance under an FA‐based surgical protocol. Therefore, the aim of this retrospective and observational study was to determine the clinical performance and complications, at a minimum 4‐ year follow‐up, in two comparable groups of patients who received a posterior stabilised (PS) TKA performed with FA by CAS and RA, respectively. It was hypothesised that a patient‐specific alignment performed with different surgical enabling technology leads to similar clinical results.

## METHODS

The present retrospective, observational study included a consecutive series of patients with end‐stage knee osteoarthritis (OA) based on the Kellgren–Lawrence classification (K‐L 3/4) treated with TKA between January 2016 and January 2020 at a single centre.

**Figure 1:**
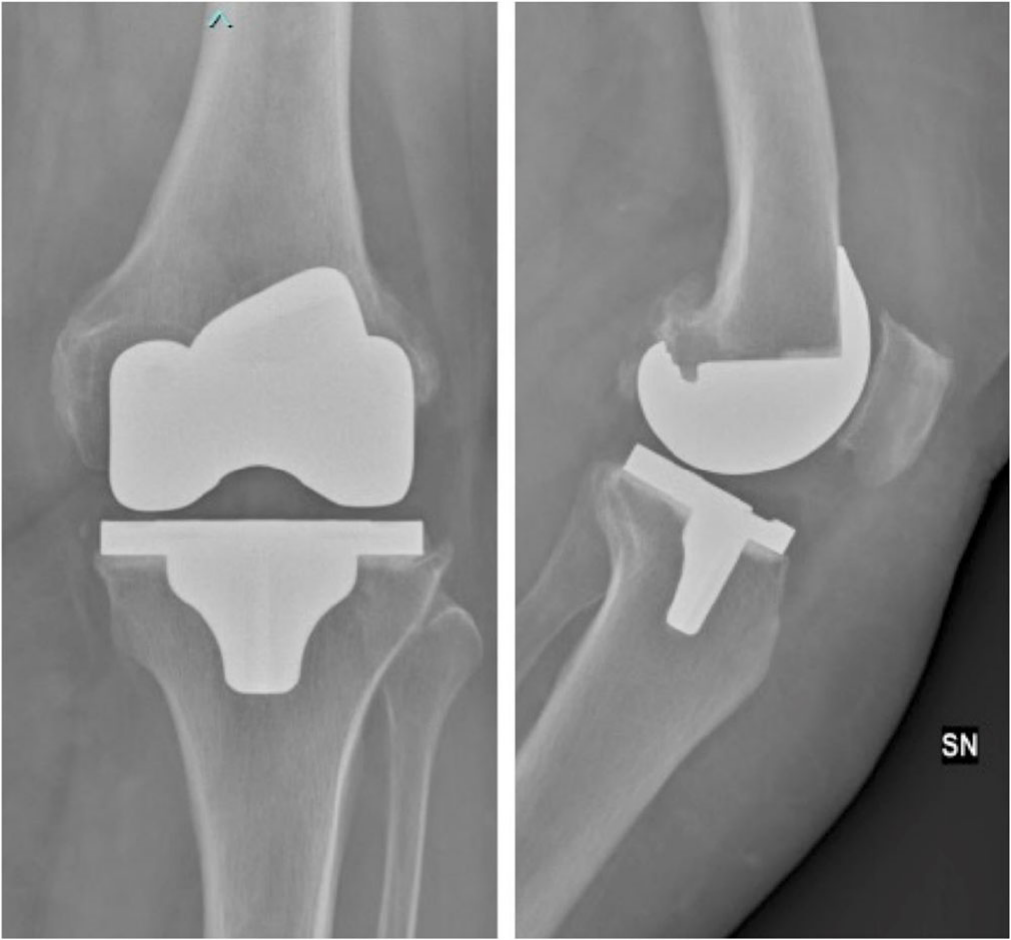
Posterior stabilised (PS) triathlon total knee implant (Stryker, Mahwah, New Jersey, USA).

All patients received a posterior stabilised (PS) triathlon total knee implant (Stryker, Mahwah, New Jersey, USA) following tibia‐based FA principles [34] (Figure 1).

Ninety‐four consecutive patients (97 knees) undergoing TKA with FA were selected for the study.

**Figure 2:**
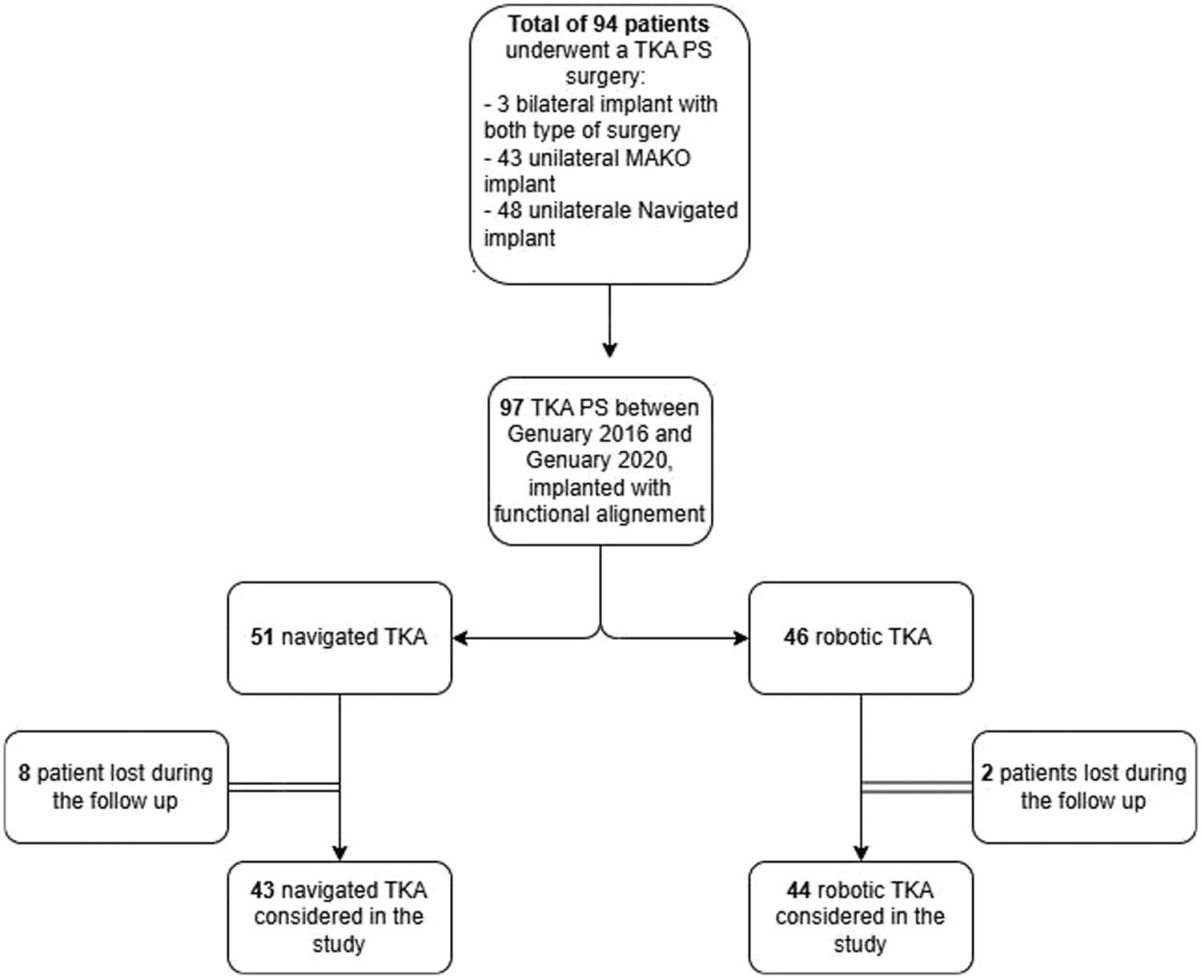
Flow chart of the inclusion process. PS, posterior stabilised; TKA, total knee arthroplasty.

Only patients undergoing cemented PS TKA performed with FA approach were included in this study. In the CAS group, although a larger number of procedures were performed during the study period, only those conducted using the FA protocol were selected (Figure 2).

Similarly, in the RA‐TKA group, only cases performed using PS implants were included. During the initial phase of the robotic programme, PS implants were adopted exclusively; as the surgeon progressed along the learning curve, cruciate‐retaining (CR) implants were increasingly used and were therefore excluded from this analysis to maintain consistency.

The two technologies were implemented sequentially throughout the study period: CAS was used almost exclusively during the initial phase, followed by a transitional period in which both CAS and RA‐TKA were employed, and finally, RA‐TKA became the predominant technique as the surgeon's experience with the robotic platform matured.

A total of 48 patients were treated with CAS (NAV 3i; Stryker Kalamazoo, MI) and 43 with robotics (Mako Surgical Corp. (Stryker), Fort Lauderdale FL, USA). Three patients underwent CAS on one side and RA‐TKA in the contralateral joint.

Patients with disabling knee pain and a radiographic diagnosis of primary OA or post‐traumatic arthritis were included for assessment. Patients with a diagnosis of inflammatory arthritis, clinical signs of collateral ligament incompetency treated with a higher level of constraint were excluded from assessment.

All surgery was performed following the same preoperative and postoperative rehabilitation protocols.

As a standard pre‐operative protocol, all patients were evaluated with the Forgotten Joint Score‐12 (FJS‐12) [3] and the Knee Injury and Osteoarthritis Outcome Score for Joint Replacement (KOOS‐JR) [20] to assess awareness and joint pain. As part of the preoperative radiographic evaluation, the medial proximal tibial angle (MPTA), lateral distal femoral angle (LDFA), Coronal Plane Alignment of the Knee (CPAK) classification and Joint Line Obliquity (JLO) were calculated for both cohorts [21]. These measurements were obtained from standardised full‐length, weight‐bearing lower limb radiographs.

The present study was performed in accordance with the Ethical Standards of the 1964 Helsinki Declaration and its later amendments and Comitato Etico Area Vasta Emilia Nord Istitutional Review Board approval was obtained (7/2024/OSS/AOUMO, transmitted with protocol N. 4527/2024).

### Surgical technique

All TKAs were performed with a standard medial parapatellar approach and only the deep fibres of the MCL were elevated, allowing better tibial exposure in varus knees. In none of the cases, the collateral ligaments were not routinely released, and the tourniquet was not used in any of the cases included. Optical tracking arrays were fixed to the bones (tibia and femur) via self‐drilling and self‐tapping pins and anatomic landmarks were identified to construct anatomic coordinate systems as per the protocol of each system.

The anterior and the posterior cruciate ligaments were resected and femoral and tibial osteophytes were removed before balancing in both the techniques.

The surgeon's preferred technique was gap‐balanced tibia‐first technique aiming for femoral component functional alignment with balanced flexion and extension gaps.

### CAS TKA

The surface of the distal femur and proximal tibia were mapped with the probe to reproduce a 3D surface. Tibial cut was performed with the CAS embedded cutting jig, considering the radiographic patient's native epiphyseal alignment (mechanical medial proximal tibial angle [MPTA]) and the tibial posterior slope, after correcting for bone wear. Tibial component ‘safe alignment’ boundaries were 4° varus to 2° valgus in the coronal plane and 0°–7° in the sagittal plane. After the tibial cut, laminar spreaders were applied in extension and at 90° of knee flexion to tension the medial and lateral extension and flexion gaps. The femoral component size, rotation and alignment were then confirmed by the surgeon performing the intraoperative plan. The effect of femoral cut adjustments was displayed on the navigation system screen. Equal flexion and extension gaps were achieved within accepted ‘safe component's alignment’ boundaries (femoral coronal alignment 5° varus to 5° valgus, femoral rotational alignment 6° internal‐6° external rotation relative to posterior condylar axis). The aim was to obtain medial and lateral symmetrical gaps in extension and flexion of approximately 18–19 mm. Femoral bone cuts were performed with a freehand saw once the cutting blocks were navigated and fixed to the defined position.

### Image‐based RA TKA

Bone registration was performed with a sharp probe capturing 40 points on the bone surface of the femur and tibia. Femoral and tibial components' coronal alignment was planned preoperatively on the preoperative CT scan, by setting equal medial and lateral bone resections, thus matching the preoperative CT‐based lateral distal femoral angle (LDFA) and MPTA, and correcting for bone wear, if present. The sagittal alignment of the tibial cut was planned according to the patient's posterior slope as visualised on the preoperative CT scan. Tibial component alignment was set within a safe zone of 4° varus to 2° valgus in the coronal plane and 0°–7° of posterior slope in the sagittal plane. The predefined alignment was reported intra‐operatively and tibial cut was performed with the haptically controlled robotic arm. Ligament tensioning was performed thereafter both in extension and at 90° of knee flexion. With ligaments tensioned, the overall limb alignment and the medial and lateral gaps were displayed on the robotic system screen. The flexion and extension gaps were then balanced by precisely adjusting the alignment of the femoral component in all three dimensions.

The target spaces in flexion and extension were the same as in the CAS technique. Femoral bone cuts were performed according to defined intraoperative plan and using the haptically controlled robotic arm. The haptic function prevents the saw from crossing cortical boundaries, helping to ensure that only planned bone is removed and to avoid inadvertent soft tissue injury.

Once bone cuts were performed, trial prosthetic components were implanted, and alignment and gaps were checked. A posterior stabilised TKA was implanted in all cases with bone cement, and patella was not routinely resurfaced.

### Data collection

All patients were followed up with postoperative periodic evaluations at 3, 6, 12 months and then annually. After a minimum two years after the surgery patients were evaluated with the FJS‐12 and the KOOS‐JR. Patients were also asked about their satisfaction with the outcomes of surgery using a 5‐point Likert scale made of five items: 'very satisfied', 'satisfied', 'neutral', 'dissatisfied', 'strongly dissatisfied'. FJS‐12, KOOS‐JR patient acceptable symptom state (PASS) [19] and minimal clinical important difference (MCID) [12, 30] values were considered based on previously published studies. Postoperative complications were recorded. Patients who did not attend follow‐up visits were contacted by phone, and if unresponsive after two attempts, were considered lost to follow‐up.

### Statistical analysis

Descriptive characteristics of the study were calculated: continuous variables were described using the mean and standard deviations, while absolute numbers and percentage were used for categorical variables. The assessment of differences between RA‐TKA and CAS groups was performed relying on parametric and non‐parametric tests, according to the adherence of the data to the test assumptions. For numerical variables, Student's t tests or Wilcoxon–Mann–Whitney tests were used, whereas Pearson's Chi squared tests or Fisher exact tests were considered for categorical variables. When the underlying assumptions were met, multivariable generalised linear regression models were also estimated, to adjust the effects of the group with respect to relevant covariates, namely the length of the follow‐up time and the baseline values of FJS and KOOS scores. The sample size was determined to guarantee a power of 90% when testing the null hypothesis of no difference among the two groups in terms of KOOS variation from pre to post intervention (ΔKOOS), against the alternative hypothesis of a clinically relevant difference occurring, using a two‐sample means Student's t test with a significance level alpha equal to 0.05. Under the null hypothesis, we expected to observe in both groups a mean ΔKOOS equal to 50, with standard deviation equal to 20. The difference in ΔKOOS between the two groups was considered clinically relevant if it exceeded a margin set equal to 14. Under these assumptions, 44 patients per group were the minimum sample size required. The sample size was determined to guarantee 90% power to detect a clinically relevant difference in KOOS‐JR improvement (ΔKOOS) between groups. No separate power analysis was conducted for the FJS‐12 outcome, which was considered a secondary endpoint.

For all analyses, the significance level was set at 0.05 and all results were reported with 95% confidence intervals. The analyses were carried out using R version 4.3.2 statistical software (The R Foundation For Statistical Computing, Vienna, Austria, 2023).

## RESULTS

Eight patients were lost to follow‐up in the CAS group, and two were deceased for reasons unrelated to surgical procedures. Two patients were lost to follow‐up in the RA‐TKA group and one deceased for reasons unrelated to surgery. Thus, a total of 84 patients (87 knees) with a mean age at surgery of 69.2 years (SD 8.4, range min. 49–max. 86) were included for assessment. A total of 40 patients (43 knees) were included in the CAS cohort and 44 patients (44 knees) were included in the RA‐TKA cohort resulting in a follow‐up rate of 83.3% and 95.3%, respectively.

**Figure 3:**
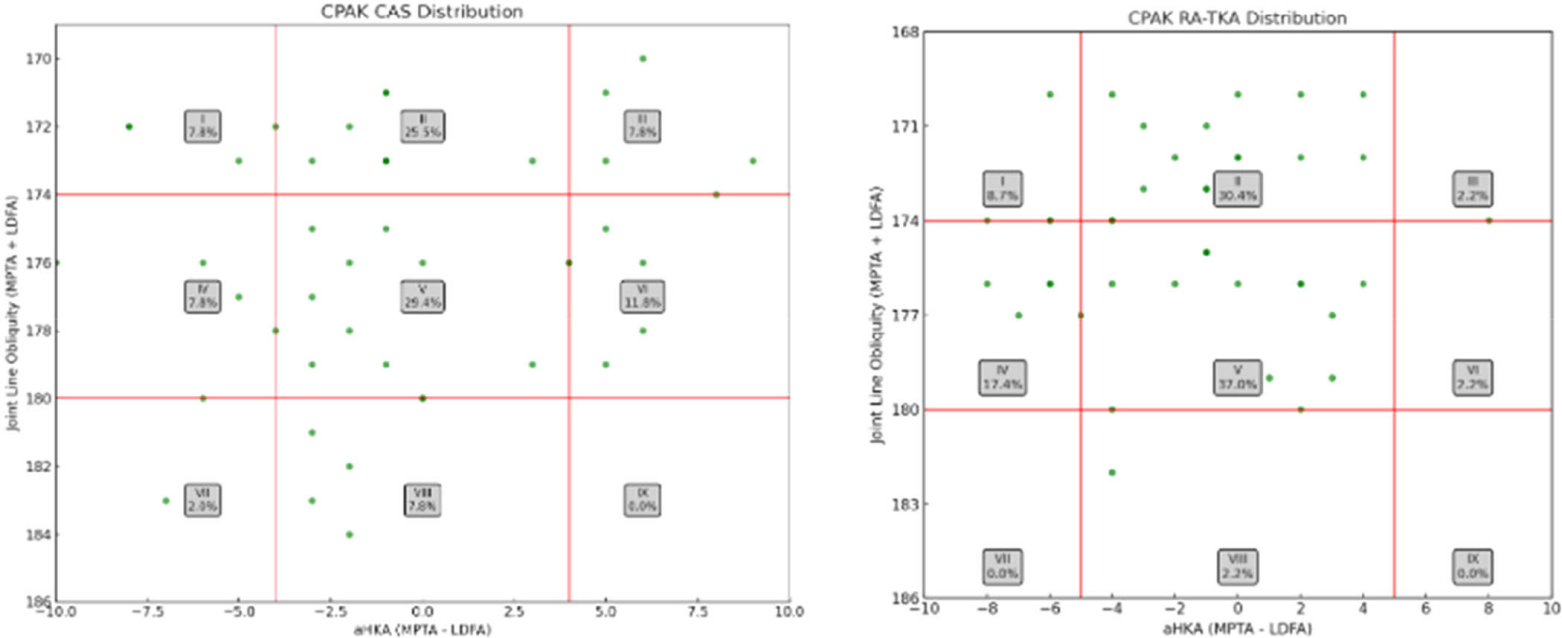
Pre‐operative CPAK full distribution CAS and RA‐TKA. CAS computer aided surgery; CPAK, coronal plane alignment of the knee; RA‐TKA, robotic assisted posterior stabilised.

**Figure 4:**
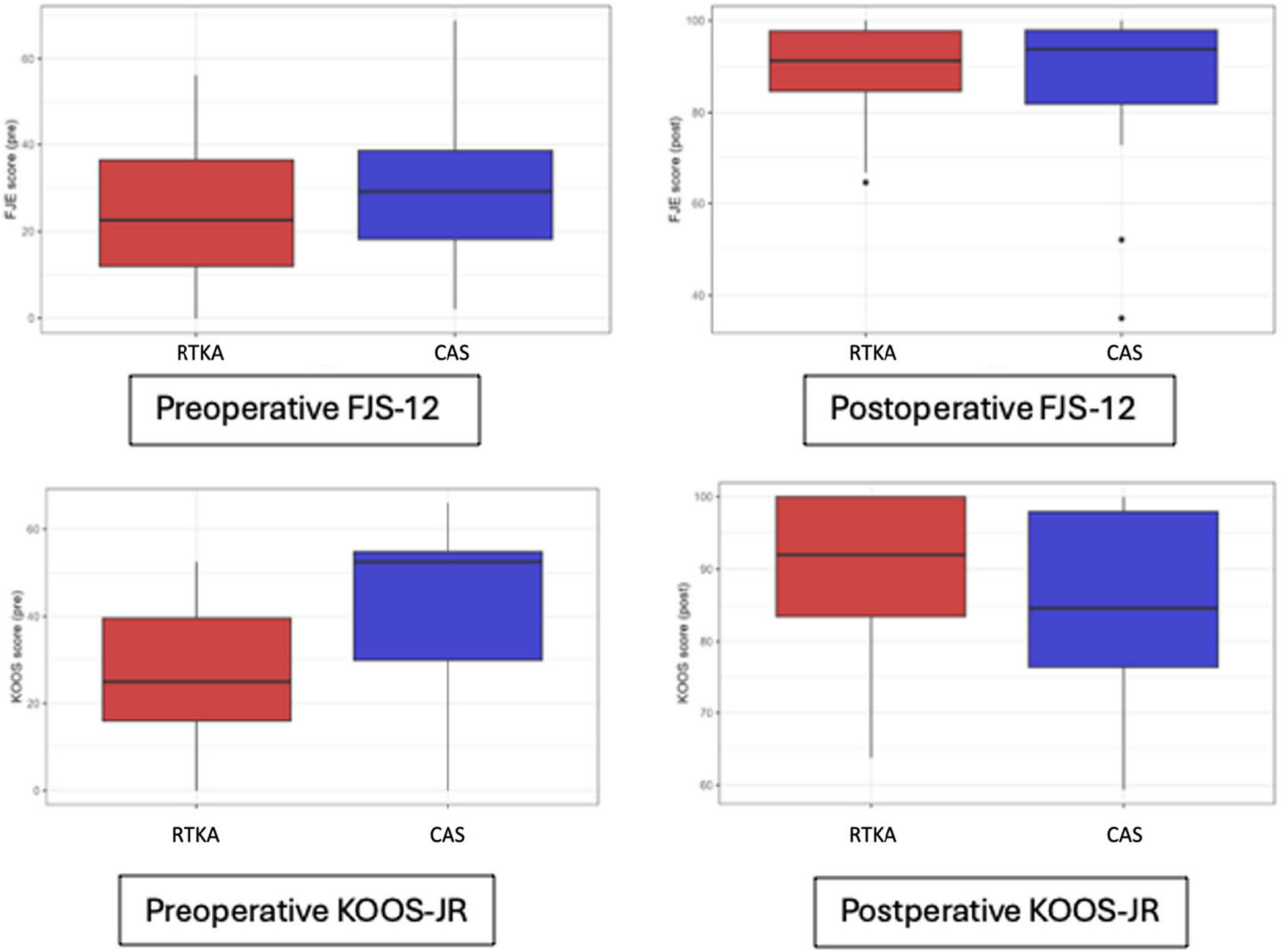
Forgotten Joint score (FJS‐12) and Knee Injury and Osteoarthritis Outcome Score for Joint Replacement (KOOS‐JR) distribution pre‐operative and post‐operative in computer aided surgery (CAS) and robotic arm assisted total knee arthroplasty (RA‐TKA).

**Figure 5:**
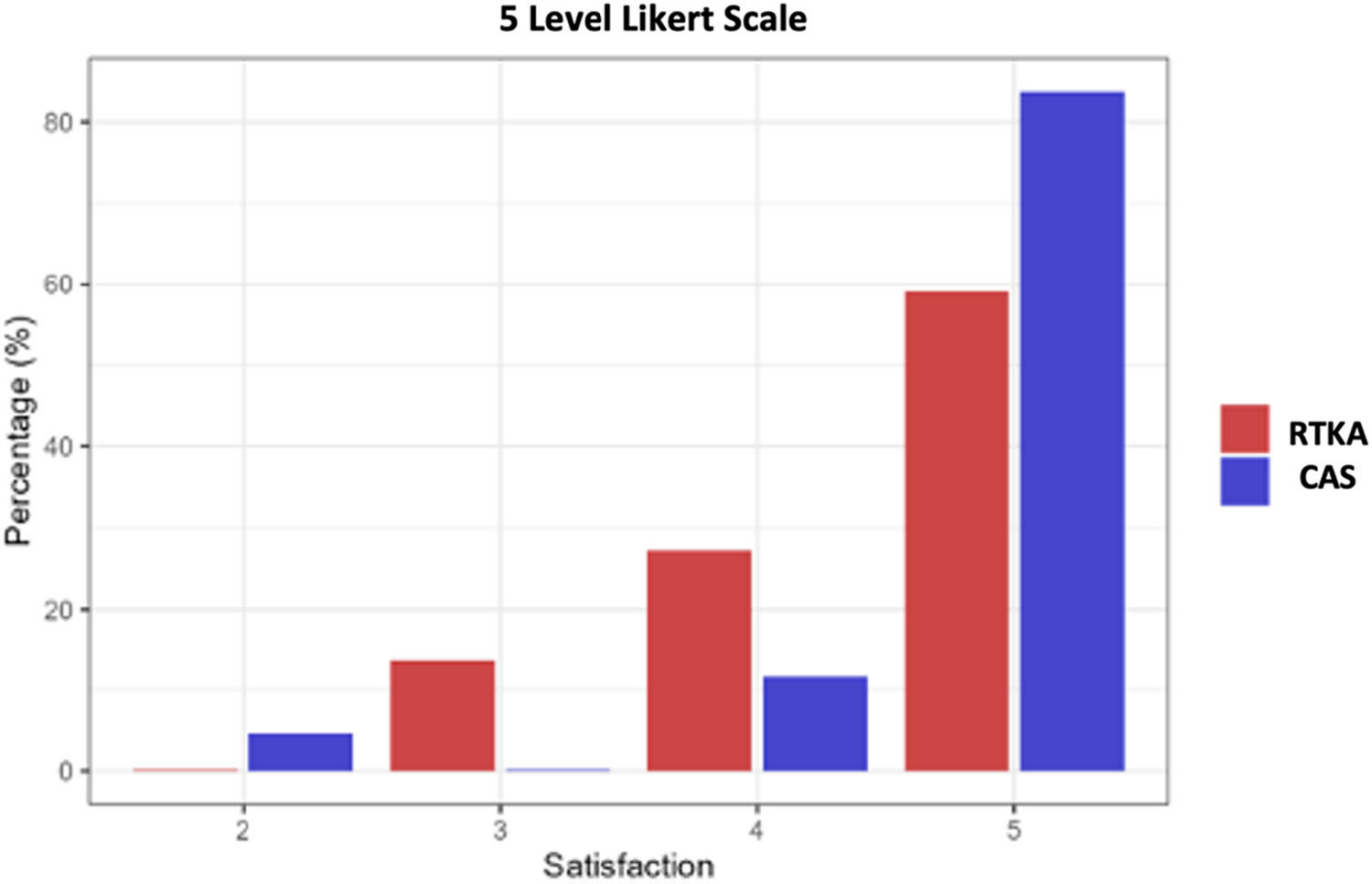
Post‐operative satisfaction distribution in RA‐TKA and CAS cohorts. CAS, computer aided surgery; RA‐TKA, robotic arm assisted total knee arthroplasty

Baseline demographic and radiographic characteristics were comparable between groups, with no statistically significant differences in age, BMI, follow‐up time, MPTA, LDFA or JLO (Table 1). Additionally, preoperative CPAK classification was calculated for all cases, and full distributions are reported in Figure 3. At baseline, the RA‐TKA group showed significantly lower KOOS‐JR scores compared to the CAS group (25.1 vs. 39.5; *p* < 0.001), while FJS‐12 scores were not significantly different (Figure 4). Patients in the CAS cohort showed a mean postoperative FJS‐12 score of 88.2 (SD 13.4) and a KOOS score of 86.2 (SD 11.5), while the RA‐TKA cohort reported a mean postoperative FJS‐12 score of 89.4 (SD 9.5) and a KOOS score of 88.0 (SD 10.3). Two patients (5%) in the CAS cohort did not reach the PASS threshold for the FJS‐12 ( > 33.7), while all patients of the RA‐TKA group reached the FJS‐12 PASS threshold; two patients in the CAS and RA‐TKA cohort (5% and 5.5%, respectively) did not reach the PASS threshold for KOOS‐JR ( > 67.7). One patient (2.3%) did not achieve the MCID for FJS‐12 (13.7) and two patients (5%) did not achieve the MCID for KOOS‐JR (14) in the CAS group when compared to the preoperative scores. All patients in the RA‐TKA achieved the MCID for FJS‐12 and KOOS‐JR. As much as 95.3% of the CAS and 86.4% of the RA‐TKA cohort were either satisfied or very satisfied with the result of the surgery, reporting a satisfaction of either 4 or 5 (Figure 5). Two patients (one per group) experienced postoperative stiffness requiring manipulation under anaesthesia; both recovered without complications. No major adverse events or revisions occurred, resulting in 100% implant survivorship in both cohorts.

**Table 1:** Demographic and radiographic characteristics of the study cohort.

Variable	RA‐TKA	CAS	Overall	*p* value
Age	69.4 ± 8.8	69.2 ± 7.7	69.3 ± 8.4	0.87
BMI	29.4 ± 5.3	29.9 ± 4.3	29.6 ± 5.0	0.58
Follow‐up	57.5 ± 7.6	67.0 ± 10.0	63.7 ± 12.6	0.20
MPTA	86.2 ± 7.8	87.5 ± 5.8	86.8 ± 7.0	0.43
LDFA	88.2 ± 6.7	87.3 ± 6.2	87.8 ± 6.5	0.54
JLO	174.5 ± 6.0	175.8 ± 7.0	175.1 ± 6.6	0.71

*Note*: Means and standard deviations are reported for different cohorts and overall group.

Abbreviations: BMI, body mass index; CAS computer aided surgery; JLO, joint line obliquity; LDFA, lateral distal femoral angle; MPTA, medial proximal tibial angle; RA‐TKA, robotic assisted posterior stabilised; SD, standard deviation.

## DISCUSSION

The most important finding of this study was the excellent clinical performance and few complications with FA in both cohorts at a minimum four‐year follow‐up. In both study groups there was an improvement from baseline conditions in terms of FJS‐12 and KOOS‐JR. Most of the patients in both groups achieved excellent satisfaction scores (89% of the sample were either 'satisfied' or 'very satisfied' for the outcome of the surgery).

The null hypothesis was confirmed as there were no statistically significant differences between the two cohort in every aspect assessed. The difference in the PASS threshold for FJS‐12 in the two cohorts may be explained by the consideration that RA‐TKA facilitating the preparation of the bone surface with the help of a robotic arm increases accuracy and reduces outliers, leading to better clinical results. As this is not a statistically significant difference (*p*‐value = 0.462) this remains only a hypothesis, further studies will be needed to validate this observation.

A possible explanation why some patients in the CAS cohort did not reach the MCID, especially for the KOOS‐JR value, may lie in the statistically significant difference between the pre‐operative KOOS‐JR values of the two groups. Although preoperative KOOS‐JR scores were slightly lower in the RA‐TKA group, indicating a more functionally compromised cohort at baseline, this difference was not statistically significant. Nevertheless, the comparable postoperative outcomes observed between the two groups may suggest a potential benefit of robotic‐assisted surgery, particularly in patients presenting with more advanced functional impairment. These findings support the hypothesis that robotic assistance may help achieve consistent results across varying preoperative conditions. It is known that the MCID is a difference between the pre‐ and post‐operative clinical status, since the CAS cohort had higher pre‐operative clinical scores, it is conceivable that the clinical difference was reduced to the point where the MCID was not reached. As, again, there were no statistically significant differences for the MCID between the two cohorts (*p* = 0.554), further studies should be awaited to understand whether the difference found in this study was due to different preoperative clinical scores or differences in the used technology.

Many studies have shown that both CAS and RA‐TKA are successful in improving alignment when compared to manual TKA technique, although these results may be biased since the comparison was made with mechanical alignment. The target of enabling technology for TKA should be considered as a ‘patient specific’ alignment [11, 31, 33], rather than a systematic approach for TKA positioning. Heckmann et al. have compared patient outcomes between CAS and conventional TKA performed with mechanical alignment and reported that the 5‐year survival of the implant was 94.7% in the conventional cohort compared with 95.1% in the computer navigated group [1]. Lee et al. also reports no statistically significant differences in mid‐ and long‐term outcomes between navigation and conventional prosthesis following mechanical alignment [18]. Nevertheless, the use of CAS has increased from 2% in the 2003 to 32% in 2019 as reported by the Australian Registry [2] and in other national registries [4].

It is realistically possible that the steady growth of robotic surgery with the development of new alignment philosophies has enabled a resurgence of navigated surgery by showing the possibility of achieving new types of alignment. This new youthfulness of navigated surgery could be attributable to the use of ‘patient‐specific’ alignment targets, and this may put it in competition with robotic surgery due also, to lower costs and a faster learning curve [7, 25]. Nevertheless, robotic surgery is becoming increasingly attractive to surgeons [22, 23], as demonstrated by data from several National Registries [2, 28]. The added prospect of precisely controlling bone cuts and the advantages of computer navigation is undoubtedly attractive to a surgeon. These advantages of using the robot maximised the benefits of using a FA and an adaptable foundation for individualised alignment. Although the CT scan is performed with the patient in a supine, non‐weight‐bearing position, image‐based robotics enable a comprehensive three‐dimensional evaluation of the joint and allow for more accurate preoperative planning by CT images, compared to the radiographic assessment which is the only performed in conventional surgery, CAS and imageless robotics [9].

Several studies have demonstrated improved clinical and functional outcomes with a technologically performed FA compared to mechanical and kinematic alignment [7, 13]. Patient‐specific alignment is guaranteed by accuracy in resections, better gap balancing and greater preservation of the patient's bone stock. In particular, the conservation of the posterior cruciate bony island may guarantee greater surgeon confidence in the use of cruciate retaining implants. This trend is increasingly observed, as evidenced by the American Joint Arthroplasty Registry in 2023 [28].

In literature there are very few studies at present comparing the clinical outcomes of the navigated and robotic technique. Ho et al. in a retrospective study report no clinical difference between the two methods using a tibia first, modified gap balancing technique at a mean follow‐up of 2 years [11]. Wang et al. in a systematic review including more than 800.000 patients who underwent total knee replacement with conventional, navigated and robotic technique at a follow‐up of 5 years showed clinical advantages in the robotic and navigated cohort compared to the conventional technique, showing no significant differences between the former two [32]. Studies with long‐term follow‐up will be necessary to assess the differences in clinical outcomes between these two technologies.

This study has several limitations. The RA‐TKA group included the surgeon's initial cases, reflecting a learning curve. Patients knew which technique was used, as this was not a blinded study. Although preoperative radiographic parameters were assessed, standardised postoperative radiographic measurements were not consistently available, limiting the author's ability to evaluate alignment correction and implant positioning. While the 4‐year follow‐up is adequate for short‐term outcome assessment, the relatively small sample size may reduce the power of survivorship and complication analyses, which should therefore be interpreted with caution. Lastly, the use of a non‐validated 5‐point Likert‐like scale for patient satisfaction introduces a potential source of bias, although it remains a widely used and pragmatic tool in clinical settings. The power analysis was based on an expected KOOS‐JR improvement of 50 points, which, although consistent with prior observations, may be influenced by inter‐individual variability and should be interpreted with caution. Although the sample size limits the statistical power of survivorship and complication analyses, the inclusion of a consecutive and homogeneous patient population with complete follow‐up still allows for meaningful preliminary observations regarding clinical safety and implant durability in both groups.

## CONCLUSIONS

The present study did not show significant differences in clinical outcomes or complication rates between patients undergoing PS‐TKA performed with CAS and those treated with RA‐TKA at a minimum four‐year follow‐up. Both techniques, when applied with a patient‐specific FA approach and a soft tissue‐sparing philosophy, resulted in excellent mid‐term outcomes, supporting the use of FA with either CAS or RA‐TKA as effective options.

## AUTHOR CONTRIBUTIONS

Stefano Seracchioli generated the hypothesis, developed the study protocol and wrote the manuscript. Francesco Zambianchi interpreted the data and drafted the manuscript. Mattia Clò collected primary data. Sebastiano Clemenza collected primary data. Riccardo Cuoghi Costantini analysed the data. Fabio Catani performed data interpretation and revised the manuscript critically.

## CONFLICT OF INTEREST STATEMENT

The authors Stefano Seracchioli, Riccardo Cuoghi Costantini, Sebastiano Clemenza and Mattia Clò, or any member of their immediate family, have no funding or commercial associations (e.g. consultancies, stock ownership, equity interest, patent/licensing arrangements, etc.) that might pose a conflict of interest in connection with the submitted article. The author Francesco Zambianchi reports speaking fees from Ab Medica S.p.A. The author Fabio Catani reports consultancy and speaking fees, royalties, and fees for participation in review activities from Stryker.

## ETHICS STATEMENT

The present study was performed in accordance with the Ethical Standards of the 1964 Helsinki Declaration and its later amendments and Comitato Etico Area Vasta Emilia Nord Istitutional Review Board approval was obtained (7/2024/OSS/AOUMO, transmitted with protocol N. 4527/2024). All patients have signed and approved the General Informed Consent prior to surgery and consented the retrospective use of intraoperative and postoperative data.

## Data Availability

The data that support the findings of this study are available from the corresponding author upon reasonable request.

## References

[1] Antonios JK , Kang HP , Robertson D , Oakes DA , Lieberman JR , Heckmann ND . Population‐based survivorship of computer‐navigated versus conventional total knee arthroplasty. J Am Acad Orthop Surg. 2020;28(20):857–864. 31934926 10.5435/JAAOS-D-19-00548

[2] Australian Orthopedic Association National Joint Replacement Registry (AOANJRR). Hip, knee and shoulder arthroplasty: annual report, Adelaide, 2020:AOA20201–474.

[3] Behrend H , Giesinger K , Giesinger JM , Kuster MS . The ‘Forgotten Joint’ as the ultimate goal in joint arthroplasty: validation of a new patient‐reported outcome measure. J Arthroplasty. 2012;27(3):430–436.e1. 22000572 10.1016/j.arth.2011.06.035

[4] Bergen H ; Norwegian Arthroplasty Register . Norwegian National Advisory Unit on arthroplasty and hip fractures 2020 annual report. Norwegian Arthroplasty Register. 2020;1:1–377.

[5] Bové JC , Clavé A . Navigated total knee arthroplasty: retrospective study of 600 continuous cases. Orthop Traumatol: Surg Res. 2021;107(3):102857. 10.1016/j.otsr.2021.102857 33588092

[6] Canovas F , Dagneaux L . Quality of life after total knee arthroplasty. Orthop Traumatol: Surg Res. 2018;104(1S):S41–S46. 29183821 10.1016/j.otsr.2017.04.017

[7] Choi BS , Kim SE , Yang M , Ro DH , Han HS . Functional alignment with robotic‐arm assisted total knee arthroplasty demonstrated better patient‐reported outcomes than mechanical alignment with manual total knee arthroplasty. Knee Surg Sports Traumatol Arthrosc. 2023;31(3):1072–1080. 36378291 10.1007/s00167-022-07227-5

[8] De Steiger RN , Liu Y‐L , Graves SE . Computer navigation for total knee arthroplasty reduces revision rate for patients less than sixty‐five years of age. J Bone Jt Surg. 2015;97:635–642. 10.2106/JBJS.M.01496 25878307

[9] Frontalis A , Luyckx T , Vanspauwen T , Moreels R , Mancino F , Raj RD , et al. Strong correlation between standing long‐leg radiographs and CT scans in measuring coronal knee alignment. J Bone Joint Surg Am. 2024;106(15):1373–1383. 38739702 10.2106/JBJS.23.01092 PMC11593997

[10] Hazratwala K , Gouk C , Wilkinson MPR , O'Callaghan W , et al. Navigated functional alignment total knee arthroplasty achieves reliable, reproducible and accurate results with high patient satisfaction. Knee Surg Sports Traumatol Arthrosc. 2023;31(9):3861–3870. 36917248 10.1007/s00167-023-07327-w PMC10435654

[11] Ho JPY , Jagota I , Twiggs J , Liu DW . Robotic‐assisted total knee arthroplasty results in shorter navigation working time similar clinical outcomes compared to computer‐navigated total knee arthroplasty. J Arthroplasty. 2024;40(4):893–899. 39307202 10.1016/j.arth.2024.09.026

[12] Holtz N , Hamilton DF , Giesinger JM , Jost B , Giesinger K . Minimal important differences for the WOMAC osteoarthritis index and the Forgotten Joint Score‐12 in total knee arthroplasty patients. BMC Musculoskelet Disord. 2020;21:401. 32576163 10.1186/s12891-020-03415-x PMC7313217

[13] Jeffrey M , Marchand P , Kouyoumdjian P , Coulomb R . Short‐term functional outcomes of robotic‐assisted TKA are better with functional alignment compared to adjusted mechanical alignment. SICOT‐J. 2024;10:2. 38240728 10.1051/sicotj/2024002 PMC10798231

[14] Jones CW , Jerabek SA . Current role of computer navigation in total knee arthroplasty. J Arthroplasty. 2018;33(7):1989–1993. 29506932 10.1016/j.arth.2018.01.027

[15] Kayani B , Konan S , Tahmassebi J , Pietrzak JRT , Haddad FS . Robotic‐arm assisted total knee arthroplasty is associated with improved early functional recovery and reduced time to hospital discharge compared with conventional jig‐based total knee arthroplasty: a prospective cohort study. Bone Joint J. 2018;100(B7):930–937. 29954217 10.1302/0301-620X.100B7.BJJ-2017-1449.R1 PMC6413767

[16] Kayani B , Konan S , Tahmassebi J , Oussedik S , Moriarty PD , Haddad FS . A prospective double‐blinded randomised control trial comparing robotic arm‐assisted functionally aligned total knee arthroplasty versus robotic arm‐assisted mechanically aligned total knee arthroplasty. Trials. 2020;21(1):194. 32070406 10.1186/s13063-020-4123-8 PMC7027302

[17] Kazarian GS , Lieberman EG , Hansen EJ , Nunley RM , Barrack RL . Clinical impact of component placement in manually instrumented total knee arthroplasty: a systematic review. Bone Joint J. 2021;103–B(9):1449–1456. 10.1302/0301-620X.103B9.BJJ-2020-1639.R2 34465158

[18] Lee D‐Y , Park Y‐J , Hwang S‐C , Park J‐S , Kang D‐G . No differences in mid‐ to long‐term outcomes of computer‐assisted navigation versus conventional total knee arthroplasty. Knee Surg Sports Traumatol Arthrosc. 2020;28(10):3183–3192. 31784782 10.1007/s00167-019-05808-5

[19] Lyman S , Lee YY , McLawhorn AS , Islam W , MacLean CH . What are the minimal and substantial improvements in the HOOS and KOOS and JR versions after total joint replacement? Clin Orthop Relat Res. 2018;476:2432–2441. 30179951 10.1097/CORR.0000000000000456 PMC6259893

[20] Lyman S , Lee YY , Franklin PD , Li W , Cross MB , Padgett DE . Validation of the KOOS, JR: a short‐form knee arthroplasty outcomes survey. Clin Orthop Relat Res. 2016;474:1461–1471. 26926773 10.1007/s11999-016-4719-1 PMC4868168

[21] Macdessi SJ , Griffiths‐Jones W , Harris IA , Bellemans J , Chen DB . Coronal plane alignment of the knee (CPAK) classification: a new system for describing knee phenotypes. Bone Joint J. 2021;103–B(2):329–337. 10.1302/0301-620X.103B2.BJJ-2020-1050.R1 PMC7954147 33517740

[22] Maman D , Laver L , Becker R , Mahamid A , Berkovich Y . Robotic‐assisted total knee arthroplasty reduces postoperative complications and length of stay without increased cost compared to navigation‐guided techniques: a national analysis. Knee Surg Sports Traumatol Arthrosc. 2025;33(1):336–342. 38953206 10.1002/ksa.12348 PMC11716347

[23] Maman D , Laver L , Becker R , Takrori LA , Mahamid A , Finkel B , et al. Trends and epidemiology in robotic‐assisted total knee arthroplasty: reduced complications and shorter hospital stays. Knee Surg Sports Traumatol Arthrosc. 2024;32(12):3281–3288. 39016343 10.1002/ksa.12353 PMC11605021

[24] Mathew K , Marchand KB , Tarazi JM , Salem HS , DeGouveia W , Ehiorobo JO , et al. Computer‐assisted navigation in total knee arthroplasty. Surg Technol Int. 2020;36:323–330. 32294224

[25] Parratte S , Van Overschelde P , Bandi M , Ozturk BY , Batailler C . An anatomo‐functional implant positioning technique with robotic assistance for primary TKA. Knee Surg Sports Traumatol Arthrosc. 2023;31(4):1334–1346. 35552475 10.1007/s00167-022-06995-4

[26] Postler A , Lützner C , Beyer F , Tille E , Lützner J . Analysis of total knee arthroplasty revision causes. BMC Musculoskelet Disord. 2018;19(1):55. 29444666 10.1186/s12891-018-1977-y PMC5813428

[27] Roof MA , Kreinces JB , Schwarzkopf R , Rozell JC , Aggarwal VK . Are there avoidable causes of early revision total knee arthroplasty? Knee Surg Relat Res. 2022;34(1):29. 35717341 10.1186/s43019-022-00157-z PMC9206343

[28] Ryan SP , Stambough JB , Huddleston JI , Levine BR . Highlights of the 2023 American Joint Replacement Registry Annual Report. Arthroplast Today. 2024;26:101325. 10.1016/j.artd.2024.101325 39006856 PMC11239969

[29] Sheridan GA , Abdelmalek M , Howard LC , Neufeld ME , Masri BA , Garbuz DS . Navigated versus conventional total knee arthroplasty: a systematic review and meta‐analysis of prospective randomized controlled trials. J Orthop. 2023;50:99–110. 38187368 10.1016/j.jor.2023.11.070 PMC10770435

[30] Singh V , Fiedler B , Huang S , Oh C , Karia RJ , Schwarzkopf R . Patient acceptable symptom state for the forgotten joint score in primary total knee arthroplasty. J Arthroplasty. 2022;37(8):1557–1561. 35346809 10.1016/j.arth.2022.03.069

[31] Varshneya K , Hong CS , Tyagi V , Ruberte Thiele RA , Huddleston JI . Imageless computer navigation reduces 5‐year all‐cause revision rates after primary total knee arthroplasty. J Arthroplasty. 2022;37(6S):S211–S215. 35256233 10.1016/j.arth.2022.02.004

[32] Wang JC , Piple AS , Hill WJ , Chen MS , Gettleman BS , Richardson M , et al. Computer‐navigated and robotic‐assisted total knee arthroplasty: increasing in popularity without increasing complications. J Arthroplasty. 2022;37(12):2358–2364. 35738360 10.1016/j.arth.2022.06.014

[33] Webb ML , Hutchison CE , Sloan M , Scanlon CM , Lee GC , Sheth NP . Reduced postoperative morbidity in computer‐navigated total knee arthroplasty: a retrospective comparison of 225,123 cases. Knee. 2021;30:148–156. 33930702 10.1016/j.knee.2020.12.015

[34] Zambianchi F , Bazzan G , Marcovigi A , Pavesi M , Illuminati A , Ensini A , et al. Joint line is restored in robotic‐arm‐assisted total knee arthroplasty performed with a tibia‐based functional alignment. Arch Orthop Trauma Surg. 2021;141:2175–2184. 34255176 10.1007/s00402-021-04039-z

